# Correction: Cost of Anti-CD38 Monoclonal Antibodies in Combination With Bortezomib, Lenalidomide and Dexamethasone for the Frontline Treatment of Transplant-Ineligible Patients With Newly Diagnosed Multiple Myeloma in the US

**DOI:** 10.36469/001c.143106

**Published:** 2025-08-12

**Authors:** Niodita Gupta-Werner, Vipin Khare, Brian Macomson, Rohan Medhekar

**Affiliations:** 1 Johnson & Johnson Innovative Medicine, Johnson & Johnson (United States)

**Keywords:** cost analysis, daratumumab, isatuximab, wholesale acquisition cost, multiple myeloma

## Abstract

**Background:** The efficacy of the combination of bortezomib, lenalidomide, and dexamethasone with daratumumab (DVRd) or isatuximab (IsaVRd) for the frontline treatment of transplant-ineligible (TIE) newly diagnosed multiple myeloma (NDMM) has been demonstrated in clinical trials. However, the treatment cost for DVRd and IsaVRd has not been compared. **Objectives:** To compare the drug acquisition costs (DAC) of DVRd vs IsaVRd in the first 2 years of frontline treatment for TIE patients with NDMM in the United States. **Methods:** Dosing schedules from the CEPHEUS and IMROZ clinical US trials were used for this analysis. AnalySource® was utilized to access the First Databank drug pricing database to collect current US DACs. Drug administration time and costs were identified and weighted, assuming 40% and 60% received the drug in a hospital outpatient and community oncology setting, respectively. Total costs were calculated by adding DACs and drug administration costs. **Results:** The DAC was 200 866inyear1and137 434 in year 2 for daratumumab and 212 421inyear1and144 143 in year 2 for isatuximab. The DAC of daratumumab was 18 264(5.417 269 and 17 327lessthanIsaVRdinpatients<75yearsand≥75yearsold,respectively.Inyear2,thetotalcostofDVRdperpatientwas10 444 and 10 553lessthanIsaVRdinpatients<75yearsand≥75yearsold,respectively.Acrossyears1and2,totalcostofDVRdperpatientwas27 713 and $27 880 less than IsaVRd in patients <75 years and ≥75 years old, respectively. Compared with isatuximab, treatment with daratumumab saves 36.13 and 22.17 hours of administration time in the first and second year, respectively. **Discussion:** This analysis shows that the DAC of DVRd is less than IsaVRd for the frontline treatment of TIE NDMM patients. DVRd results in time savings vs IsaVRd, which is preferable for patients and caregivers. **Conclusions:** DVRd is a timesaving and less expensive frontline treatment option for patients with TIE NDMM than IsaVRd in the first and second year of treatment.

## INTRODUCTION

Multiple myeloma (MM) is a rare hematological malignancy with a global incidence of 1.78 per 100 000 in 2020.^1^ Despite its rarity, MM poses a substantial burden due to the frequency of relapse and the need for multiple treatment regimens.^2^ Treatment of patients with newly diagnosed multiple myeloma (NDMM) depends on their eligibility for stem cell transplantation, which is determined by assessing patient and disease characteristics, with patients who are frail or medically unfit generally deemed to be transplant-ineligible (TIE).^3,4^

Recently, a quadruplet regimen of the anti-CD38 antibody isatuximab in combination with bortezomib, lenalidomide, and dexamethasone (IsaVRd) has been approved for the treatment of TIE patients with NDMM, based on the results of the IMROZ trial.^5^ In IMROZ, the estimated percentage of patients with progression-free survival at 60 months was higher in patients treated with IsaVRd than in patients treated with VRd (hazard ratio [HR]: 0.60 [95% confidence interval [CI]: 0.41, 0.88]; *P*<.001).^5^

A quadruplet regimen of subcutaneous (SC) daratumumab in combination with bortezomib, lenalidomide, and dexamethasone (DVRd) has also been investigated as a frontline treatment option for TIE patients with NDMM in the ongoing CEPHEUS trial.^6^ Primary results of CEPHEUS showed that 60.9% of patients receiving DVRd achieved minimal residual disease negativity, compared with 39.4% of patients receiving VRd (odds ratio: 2.37 [95% CI: 1.58, 3.55]; *P*<.0005).^6^ Additionally, progression-free survival was significantly improved in patients treated with DVRd compared with VRd (HR: 0.57 [95% CI: 0.41, 0.79]; *P*=.0005).^6^ These results have been submitted to the US Food & Drug Administration.

While the efficacy of IsaVRd and DVRd have been studied in their respective clinical trials, the cost of treating patients with these regimens in the United States (US) has not been studied. Therefore, this analysis sought to assess the cost of frontline treatment with DVRd cvs IsaVRd in TIE patients with NDMM in the US, utilizing dosing schedules from CEPHEUS and IMROZ trials for the first 2 years of treatment.

## METHODS

A cost analysis was conducted to estimate the anti-CD38 agent (daratumumab or isatuximab) and total regimen cost of DVRd compared with IsaVRd from a USA payer perspective, over the first 2 years of treatment in TIE patients with NDMM.

### Drug Acquisition Costs

Dosing schedules from the CEPHEUS and IMROZ trials (**Supplementary Table S1**) were used calculate the cost of treatment with DVRd and IsaVRd regimens in TIE patients with NDMM.^6,7^ The number of doses of drugs in each regimen administered in year 1 and 2 of treatment according to the CEPHEUS and IMROZ dosing schedules are summarized in Table 1.^6,7^ In the CEPHEUS and IMROZ trials, older patients were given a lower dose/lower dosing frequency of dexamethasone (**Supplementary Table S1**). Specifically, per CEPHEUS trial design, patients that were >75 years old or with a BMI of <18.5, and per the IMROZ trial design patients that were ≥75 years old, were given a lower dose/lower dosing frequency of dexamethasone, therefore for the current cost calculation we assessed the costs separately for the two age groups. For ease of reporting, the groups were categorized as “age <75 years” and “age ≥75 years.”

The analytic tool AnalySource® was utilized to access the First Databank drug pricing database to collect information on the current (as of February 12, 2025) drug acquisition cost (DAC) of the drugs in both regimens.^8^ Daratumumab and isatuximab are available as branded products. Daratumumab SC is the standard formulation used in clinical practice and was used in the CEPHEUS trial; accordingly, DAC for Darzalex Faspro® (Janssen Biotech, Inc.) and Sarclisa® (Sanofi-Aventis U.S., LLC) were used in the current analysis.

Daratumumab SC has a fixed dose of 1800 mg, the current DAC for which is $10 572.^8,9^ The current DAC for isatuximab is $843 and $4215 for 100 mg and 500 mg vials, respectively.^8^ The recommended dose for isatuximab is 10 mg/kg of body weight and is administered as an intravenous (IV) injection.^10^ The median patient weight at MM diagnosis is reported to be approximately 82 to 85 kg.^11,12^ Thus, the required dose of isatuximab was estimated to be 820 to 850 mg. Drug wastage was accounted for in this calculation, therefore one 500 mg vial and four 100 mg vials of isatuximab would be required per dose which would result in a DAC of $7586. This cost remains valid for any patient with body weight >80 kg and ≤90 kg. However, there is the possibility that two 500 mg vials would be used, with a wastage of 100 mg, which would result in an even higher DAC for isatuximab. This scenario has not been considered for this analysis.

The recommended dose for bortezomib is 1.3 mg/m^2^ of body surface area, mostly administered via SC injection and is available as a 3.5 mg single-use vial. Bortezomib is available as a generic, so the DAC of $35 for the lowest cost generic is used in this model.^8^ Lenalidomide is administered orally and is partly available as a generic drug, however, most lenalidomide use in the US is currently branded (Revlimid®, Bristol Myers Squibb) with a DAC of $892, which was used in this model.^8^

In both clinical trials, dexamethasone could be administered either orally or intravenously.^6,7^ For the purpose of this analysis, dexamethasone was assumed to be administered orally. Dexamethasone is a commonly available generic drug, with a DAC of $1 for 6 mg for oral dexamethasone and $0.45 for 4 mg oral dexamethasone.^8^ Using these prices, the DACfor 20 mg and 40 mg of oral dexamethasone is calculated at $2 and $4, respectively.

### Drug Administration Costs

Administration time and costs were considered for daratumumab and isatuximab only in this analysis, as lenalidomide is an oral drug and, as previously mentioned, dexamethasone was assumed to be administered orally. Although bortezomib is administered subcutaneously, the number of doses and administration time is the same in both DVRd and IsaVRd regimens (**Supplementary Table S1**), hence administration time and cost of bortezomib was not included in this analysis. In our model, we assumed that 40% of patients received their drug administration in a hospital outpatient setting, while 60% have their drugs administered in a community oncology setting.

The infusion time for IV isatuximab is 3.3 hours for the first administration, 1.9 hours for the second administration, and 1.3 hours for subsequent administrations.^13^ While the time taken for administration of daratumumab via SC injection is much shorter, at approximately 3 to 5 minutes.^14^ Drug administration costs were calculated using the Centers for Medicare & Medicaid Services estimates for IV or SC administration at hospital outpatient or community oncology clinic settings.^15,16^

## Total Costs of Treatment

The total cost of treatment per patient for DVRd and IsaVRd regimens for the first 2 years of treatment was calculated as the sum of the DAC of each drug per the dosing schedule from each respective trial and the administration costs of each drug.

## RESULTS

### Drug Acquisition Costs

The DAC of DVRd and IsaVRd regimens and each individual drug in these regimens, based on the dosing schedule used in the CEPHEUS and IMROZ trials, are summarized in **Table 1**. With respect to the anti-CD38 agent cost, the DAC of daratumumab SC was $11 555 less in the first year of treatment, and $6709 less in the second year of treatment compared with the DAC of isatuximab. Across the first 2 years of treatment, the cumulative DAC was $338 300 for daratumumab SC and $356 564 for isatuximab (**Table 1**), indicating $18 264 (4.4%) lower cost for daratumumab SC compared with isatuximab.

**Table 1.** Number of Doses, DAC, and Administration Cost of DVRd and IsaVRd

	**DVRd**		**IsaVRd**	
**No. of doses**	**Year 1**	**Year 2**	**Year 1**	**Year 2**
Anti-CD38 mAb^a^	19	13	28	19
Bortezomib SC	32	0	32	0
Lenalidomide	259	273	259	273
Dexamethasone (age <75 y)	92	52	96	52
Dexamethasone (age ≥75 y)	60	52	64	52
**DAC of each drug per dosing schedule ($)**	**Year 1**	**Year 2**	**Year 1**	**Year 2**
Anti-CD38 mAb^a^	200 866	137 434	212 421	144 143
Bortezomib SC	1120	–	1120	–
Lenalidomide	230 912	243 393	230 912	243 393
Dexamethasone (age <75 y)	252	218	202	109
Dexamethasone (age ≥75 y)	126	109	134	109
**Total DAC for the regimen ($)**	**Year 1**	**Year 2**	**Year 1**	**Year 2**
Age <75 y	433 149	381 046	444 655	387 646
Age ≥75 y	433 023	380 937	444 587	387 646
**Cost of drug administration^b^ ($)**	**Year 1**	**Year 2**	**Year 1**	**Year 2**
Anti-CD38 mAb^a^	1330	910	7093	4754
Bortezomib SC	2240	–	2240	–
**Total cost of treatment (total DAC for regimen + cost of drug administration) ($)**	**Year 1**	**Year 2**	**Year 1**	**Year 2**
Age <75 y	436 719	381 956	453 988	392 400
Age ≥75 y	436 593	381 847	453 920	392 400
**Cumulative total cost of treatment (2-year) ($)**	**Year 1 + Year 2**		**Year 1 + Year 2**	
Age <75 y	818 675		846 388	
Age ≥75 y	818 440		846 320	

Abbreviations: DAC, drug acquisition costs; DVRd, daratumumab in combination with bortezomib, lenalidomide, and dexamethasone; IsaVRd, isatuximab in combination with bortezomib, lenalidomide, and dexamethasone; mAb, monoclonal antibody.

^a^The brand products used for this analysis were Darzalex Faspro® (daratumumab SC; Janssen Biotech, Inc.) and Sarclisa® (isatuximab; Sanofi-Aventis U.S., LLC).

^b^Other agents that make up the quadruplet regimen are either administered orally or do not cost substantial dollar amounts for administration.

At the regimen level, the cumulative DAC across year 1 and 2 of treatment per patient totaled $814 195 for DVRd and $832 301 for IsaVRd regimens in patients with age <75 years (**Table 1**). Thus, the DVRd regimen costs $18 106 less than the IsaVRd regimen in this age group over the first 2 years of treatment. For patients with age ≥75 years, cumulative DAC across year 1 and 2 was $813 960 for DVRd regimen and $832 233 for IsaVRd regimen (**Table 1**), thus, the DVRd regimen costs $18 273 less than IsaVRd regimen in this age group.

### Drug Administration Costs

A greater number of doses is required for patients receiving isatuximab compared with patients receiving daratumumab SC in the first 2 years of treatment (**Table 1**). This in turn increases the number of clinic visits for drug administration for the patients receiving an isatuximab or IsaVRd regimen.

Using the IMROZ dosing schedule, the total time for administration of isatuximab per patient in year 1 was estimated to be 37.72 hours. While for daratumumab SC, per the CEPHEUS dosing schedule, the total time of drug administration per patient in year 1 was estimated to be 1.58 hours. Overall, the use of daratumumab SC saves 36.13 hours (~1.5 days) of drug administration time in year 1 per patient, compared with isatuximab. In year 2; total time for administration of isatuximab is estimated to be 23.75 hours, while total time for administration of daratumumab SC is estimated to be 1.58 hours. Overall, treatment with daratumumab SC saves 22.17 hours (~0.9 days) of drug administration time per patient compared with isatuximab in the second year of treatment.

Using the time taken for drug administration and the weighted administration cost (**Table 2**), the total cost of administration for each treatment regimen was calculated. The total cost of administration for isatuximab was $337 for first administration and $250 for second and subsequent administrations. The weighted cost of SC administration of daratumumab SC and bortezomib was $70.

**Table 2.** Administration Cost for Procedure

**Administration Cost for Procedure**	**Hospital Outpatient^15,16^**		**Community Oncology Clinic^16^**		**Cost Weighted per Market Share of 40% Hospital Outpatient and 60% Community Oncology Clinic**
	**APC Code**	**Unit Cost**	**CPT Code**	**Unit Cost**	
Chemo IV 1st hour	5694	$323	96413	$129	$207
Chemo IV, subsequent hours after 1st hour	5692	$67	96415	$28	$44
Chemo SC	5692	$67	96401	$72	$70

Abbreviations: APC, ambulatory payment classification; CPT, current procedural terminology; IV, intravenous; SC, subcutaneous.

Administration costs are from 2024 and are reported in US dollars.

Based on the drug administration prices weighted for a clinical setting, the administration cost of DVRd and IsaVRd per the dosing schedule are summarized in **Table 1**. With respect to the anti-CD38 agent administration cost, in the first year of treatment, daratumumab SC had a $5763 lower cost of administration than isatuximab. In the second year of treatment, daratumumab SC had $3844 lower cost of administration than isatuximab. At the regimen level, the cumulative cost of drug administration across year 1 and 2 of treatment totaled to $4480 for DVRd regimen, while that for IsaVRd regimen totaled to $14 087 (**Table 1**). Thus, the DVRd regimen costs $9607 less to administer than the IsaVRd regimen over the first 2 years of treatment.

### Total Cost of Treatment

The total cost of treatment (DAC + administration cost) per patient for each regimen in the first 2 years of treatment are described in **Table 1**. In year 1, the cost of DVRd was $17 269 and $17 327 less than IsaVRd in patients <75 years old and ≥75 years old, respectively. In year 2, DVRd cost $10 444 less than IsaVRd in patients <75 years old and $10,553 less than IsaVRd in patients ≥75 years old. Across years 1 and 2, DVRd cost $27 713 less than IsaVRd in patients <75 years old and $27 880 less than IsaVRd in patients ≥75 years old.

## DISCUSSION

This analysis evaluated, for the first time, the estimated cost of DVRd compared with IsaVRd in TIE patients with NDMM from a US payer’s perspective. When utilizing the dosing schedules used in CEPHEUS and IMROZ, this analysis demonstrated that daratumumab SC has a lower cost than isatuximab in terms of drug administration costs and total cost per patient in both the first and second year of treatment. Overall, this study shows that DVRd is a less costly frontline quadruplet treatment for TIE patients with NDMM compared with IsaVRd in the first 2 years of treatment.

Per the CEPHEUS and IMROZ trials, daratumumab is delivered subcutaneously, while isatuximab is delivered intravenously. This analysis showed that the use of daratumumab SC reduced the duration of drug administration per patient in years 1 and 2, compared with isatuximab. The use of SC over IV administration has been shown to be beneficial, as it is more convenient and reduces burden on patients and healthcare providers.^17^ Consequently, it has been shown that patients have a strong preference for SC over IV delivery.^18^ In addition, patients with cancer have shown a strong preference for receiving drugs with shorter infusion times, as they cause less disruption to their daily life.^19^ It has been shown that direct and indirect costs, such as healthcare resource utilization and medical personnel time, are higher for regimens that require more frequent or longer duration of drug administration.^20^ Therefore, the time saved when administering daratumumab SC compared with isatuximab IV may also impact the total cost of treatment, beyond the DAC of the individual drugs.

While generic bortezomib and branded lenalidomide were considered in this calculation, the overall cost of these agents does not affect the overall cost difference among the two regimens, as the dosing schedule and the costs for these agents is the same for both the regimens.

In this analysis, a 2-year timeframe was selected as it could be more relevant for payers as it would allow them to focus on the immediate and longer-term costs associated with treatments, with consideration of their current budget, changing drug costs, and patient turnover.^21^

A limitation of this study is that only medication and administrations costs of the studied regimens were considered, while clinic time and other healthcare utilization costs, such as doctor visits and emergency room visits, were not included in this analysis. However, the real-world healthcare resource use and cost data are currently not available for either of these regimens. Another limitation of this study is that the impact of factors such as longer time horizon, variation in patient weight, comparative efficacy and safety of the two regimens, and the cost of managing treatment-related adverse events were not analyzed. However, this was beyond the scope of the current study and represents an opportunity for future research. The intent of this analysis was to compare the 2-year DAC and administration cost for DVRd and IsaVRd as frontline treatment for TIE patients with NDMM from a US payer perspective.

This analysis shows that DVRd is less costly than IsaVRd as frontline treatment of TIE patients with NDMM in the first 2 years of treatment. Daratumumab SC in DVRd regimen has lower dosing frequency and takes less time to administer than the isatuximab/IsaVRd regimen, which will have a positive impact on patient’s daily lives and could improve patient and caregiver satisfaction. The shorter drug administration times with DVRd compared with IsaVRd could also help to increase the productivity of healthcare professionals by enabling efficient outpatient treatment scheduling and thus, the treatment of more patients. Overall, treatment with DVRd is more time efficient and has lower costs than IsaVRd.

## Supplementary Material

Online Supplementary Material
