# Supplementary material for: Cost of Anti-CD38 Monoclonal Antibodies in Combination With Bortezomib, Lenalidomide and Dexamethasone for the Frontline Treatment of Transplant-Ineligible Patients With Newly Diagnosed Multiple Myeloma in the US
Source: J Health Econ Outcomes Res. 2025 Jul 21;12(2):27–31. doi: 10.36469/001c.141714 (PMC12285652; doi:10.36469/001c.141714)
Supplement: Online Supplementary Material [file jheor_2025_12_2_141714_294932.pdf]

Journal of Health Economics and Outcomes Research

Online Supplementary Material

Cost of Anti-CD38 Monoclonal Antibodies in Combination With Bortezomib, Lenalidomide and Dexamethasone for the Frontline Treatment of Transplant Ineligible Patients With Newly Diagnosed Multiple Myeloma in the US. *JHEOR*. 2025;12(2):27-31. doi:10.36469/jheor.2025.141714

Table S1: Dosing Schedules Used in the CEPHEUS and IMROZ Clinical Trials

This supplementary material has been provided by the authors to give readers additional information about their work.

This is an open-access article distributed under the terms of the Creative Commons Attribution 4.0 International License (CCBY-4.0). View this license's legal deed at http://creativecommons.org/licenses/by/4.0 and legal code at http://creativecommons.org/licenses/by/4.0/legalcode for more information.

Table S1. Dosing Schedules Used in the CEPHEUS and IMROZ Clinical Trials[6,7]

**IMROZ**	**Induction phase: Cycle 1-4 (6-week cycles)**																				
	Day	1	2	4	5	8	9	11	12	14	15	21	22	23	25	26	29	30	32	33	42
	Isatuximab (10 mg/kg)	X				X[a]					X		X[a]				X				
	Bortezomib (1.3 mg/m^2)	X		X		X		X					X		X		X		X		
	Lenalidomide (25 mg)[b]	Days 1-14											Days 22-35								
	Dexamethasone (20 mg)	X[c]	X	X[c]	X	X[c]	X	X[c]	X		X[c]		X[c]	X	X[c]	X	X[c]	X	X[c]	X	
	**Continuous phase: Cycle 5 onward (4-week cycles) until the occurrence of disease progression, an unacceptable adverse event, or other discontinuation criteria**																				
	Isatuximab (10 mg/kg), cycle 5-17	X									X										
	Isatuximab (10 mg/kg), cycle 18+	X																			
	Lenalidomide (25 mg)[b]	Days 1-21																			
	Dexamethasone (20 mg)	X				X					X		X								
**CEPHEUS**	**Cycle 1-8 (3-week cycles)**																				
	Day	1	2	4	5	8	9	11	12	14	15	21	22	23	25	26	29	30	32	33	42
	Daratumumab (1800 mg), cycles 1-2	X				X					X										
	Daratumumab (1800 mg), cycles 3-8	X																			
	Bortezomib (1.3 mg/m^2)	X		X		X		X													
	Lenalidomide (25 mg)	Days 1-14																			
	Dexamethasone (20 mg)	X[d]	X	X[d]	X	X[d]	X	X[d]	X												
	**Continuous phase: Cycle 8 onward (4-week cycles) until progression or unacceptable toxicity**																				
	Daratumumab (1800 mg)	Once every 4 weeks																			
	Lenalidomide (25 mg)	Days 1-21																			
	Dexamethasone (40 mg)	X[e]				X[e]					X[e]		X[e]								

[a]Cycle 1 only.
[b]10 mg per day if the estimated glomerular filtration rate was 30 to <60 ml per minute per 1.73 m^2.
[c]Patients ≥75 years receive dexamethasone only on these days.
[d]Patients >75 years or body mass index <18.5 km/m^2 receive dexamethasone only on these days.
[e]20 mg for patients >75 years or body mass index <18.5 km/m^2.
